# Molecular epidemiology of Avian Rotaviruses Group A and D shed by different bird species in Nigeria

**DOI:** 10.1186/s12985-017-0778-5

**Published:** 2017-06-12

**Authors:** Maude Pauly, Oluwole O. Oni, Aurélie Sausy, Ademola A. Owoade, Christopher A. O. Adeyefa, Claude P. Muller, Judith M. Hübschen, Chantal J. Snoeck

**Affiliations:** 1 Infectious Diseases Research Unit, Department of Infection and Immunity, Luxembourg Institute of Health, 29 rue Henri Koch, L-4354 Esch-sur-Alzette, Luxembourg; 2 Department of Veterinary Medicine and Surgery, College of Veterinary Medicine, Federal University of Agriculture, Abeokuta, Ogun State Nigeria; 3 Department of Veterinary Medicine, University of Ibadan, Ibadan, Oyo State Nigeria

**Keywords:** Avian rotaviruses, Host permissiveness, Virus diversity, Epidemiology, Sub-Saharan Africa

## Abstract

**Background:**

Avian rotaviruses (RVs) cause gastrointestinal diseases of birds worldwide. However, prevalence, diversity, epidemiology and phylogeny of RVs remain largely under-investigated in Africa.

**Methods:**

Fecal samples from 349 birds (158 symptomatic, 107 asymptomatic and 84 birds without recorded health status) were screened by reverse transcription PCR to detect RV groups A and D (RVA and RVD). Partial gene sequences of VP4, VP6, VP7 and NSP4 for RVA, and of VP6 and VP7 for RVD were obtained and analyzed to infer phylogenetic relationship. Fisher’s exact test and logistic regression were applied to identify factors potentially influencing virus shedding in chickens.

**Results:**

A high prevalence of RVA (36.1%; 126/349) and RVD (31.8%; 111/349) shedding was revealed in birds. In chickens, RV shedding was age-dependent and highest RVD shedding rates were found in commercial farms. No negative health effect could be shown, and RVA and RVD shedding was significantly more likely in asymptomatic chickens: RVA/RVD were detected in 51.9/48.1% of the asymptomatic chickens, compared to 18.9/29.7% of the symptomatic chickens (*p* < 0.001/*p* = 0.01). First RVA sequences were obtained from mallard ducks (*Anas platyrhynchos*) and guinea fowls (*Numida meleagris*). Phylogenetic analyses illustrated the high genetic diversity of RVA and RVD in Nigerian birds and suggested cross-species transmission of RVA, especially at live bird markets. Indeed, RVA strains highly similar to a recently published fox rotavirus (RVA/Fox-tc/ITA/288356/2011/G18P[17]) and distantly related to other avian RVs were detected in different bird species, including pigeons, ducks, guinea fowls, quails and chickens.

**Conclusion:**

This study provides new insights into epidemiology, diversity and classification of avian RVA and RVD in Nigeria. We show that cross-species transmission of host permissive RV strains occurs when different bird species are mixed.

**Electronic supplementary material:**

The online version of this article (doi:10.1186/s12985-017-0778-5) contains supplementary material, which is available to authorized users.

## Background

Rotavirus (RV) infections are highly prevalent worldwide [1] and cause gastroenteritis mainly in infants [2] and young animals (reviewed in [3]). Different bird species are susceptible to RV infections ([4]; reviewed in [5]). Although avian RVs are involved in the pathogenesis of the runting-stunting syndrome of broiler chicks [6], RV shedding from asymptomatic birds has also been reported [7].

The genome of these non-enveloped, double-stranded RNA viruses is composed of 11 segments, encoding six viral structural proteins (VP1 to VP4, VP6, and VP7) and five to six nonstructural proteins (NSP1 to NSP5/NSP6). Eight RV groups A to H (RVA-RVH) are defined by the International Committee on Taxonomy of Viruses (ICTV) based mainly on group-specific antigenicity of VP6 (reviewed in [8]). In addition, a percentage cut-off value for tentative group discrimination based on the amino acid sequence similarity of VP6 has been proposed [9]. Recently, putative new RV species were detected in dogs, cats and bats [10–12]. Groups A, D, F and G have been found in birds [1], with a predominance of RVA and RVD shedding [13].

Different mechanisms drive diversity of RVs: point mutations, interspecies transmissions, genetic reassortments and recombinations [14, 15]. However, virus-host coevolution remains the major evolutionary pattern reported for RVs [16] and reassortment events mainly occur between strains of the same RV group [17, 18]. Nevertheless, some natural reassortants show characteristics suggestive of mixed host species origins [19] and a potential for cross-group reassortment was revealed for RVA and RVD [20]. As previously shown for a RVD strain [20], typing based only on a few genome segments can be misleading. Thus, definitive RV type characterization necessitates whole genome sequencing [19, 21–23]. The Rotavirus Classification Working Group (RCWG) proposed a uniforme nomenclature for RVA strains based on nucleotide identity cut-off values and allowing to determine the genotype of each genome segment [24].

The prevalence, phylogenetic relation and clinical importance of avian RVs have been described for chickens and turkeys in numerous countries [13, 25–28], but rarely in Africa where the virus may cause dramatic economic losses in particular for subsistence farmers. Avian RVA shedding by diarrheic domestic poultry in Nigeria has been reported for the first time in 2010 [29], without genetic information. Although it is suspected that other poultry species are susceptible to RVs, there is only limited evidence so far. Cross-species transmission may occur when different bird species intermingle, e.g. in backyard farms and live bird markets. Here we investigated genetic diversity of RVA as well as RVD strains circulating in Nigeria to understand transmission pathways and host range.

## Methods

### Sample collection

In 2011 and 2013, fecal samples (*n* = 349) were collected from domestic birds in Ogun and Oyo States, Southwestern Nigeria (Table 1). The samples were collected from different farm types (classified as backyard farms, Farm type 1: farms with less than 500 birds, Farm type 2: farms with between 500 and 10,000 birds, and Farm type 3: farms with over 10,000 birds) and at live bird markets (Table 1). The collection sites (*n* > 40) were randomly selected to represent the avian RV situation within the different farm types. In Oyo state, no birds were sampled in backyard farms. Besides chickens (*Gallus gallus domesticus; n* = 255), ducks (*Anas platyrhynchos; n* = 24), guinea fowls (*Numida meleagris; n* = 25), pigeons (*Columba livia; n* = 19), quails (*Coturnix coturnix; n* = 16) and turkeys (*Meleagris gallopavo*; *n* = 10) were included. In 2011, age and health status of the flocks were recorded. In 2013, various bird species were sampled irrespective of their health status.

**Table 1:** Demographic characteristics and rotavirus A (RVA) and D (RVD) shedding

Sample information	Dataset		Rotavirus positivity			
			RVA		RVD	
	N	%	n	%	n	%
Year of sample collection						
2011	199	57	59	29.6	82	41.2
2013	150	43	67	44.7	29	19.3
Observed symptoms						
Diarrhea or increased mortality	158	45.3	29	18.4	45	28.5
None	107	30.7	57	53.3	42	39.3
No information	84	24.1	40	47.6	24	28.6
Age group						
1 (1–25 days)	76	21.8	23	30.3	22	28.9
2 (26–140 days)	117	33.5	42	35.9	63	53.8
3 (>140 days)	156	44.7	61	39.1	26	16.7
Species						
Chicken	255	73.1	82	32.2	92	36.1
Other	94	26.9	44	46.8	19	20.2
State						
Ogun	249	71.3	99	39.8	70	28.1
Oyo	100	28.7	27	27	41	41
Collection site						
Backyard farm	18	5.2	5	27.8	5	27.8
Farm 1 (<500 animals)	60	17.2	14	23.3	6	10
Farm 2 (500 ≤ x ≤ 10,000 animals)	99	28.4	32	32.3	36	36.4
Farm 3 (>10,000 animals)	95	27.2	31	32.6	49	51.6
Live bird market	77	22.1	44	57.1	15	19.5

Samples were directly placed on ice, transported to the laboratory and preserved at −20 °C until shipment to Luxembourg, where they were stored at −80 °C until further processing.

### RNA extraction

Feces were resuspended in 500 μl of virus transport medium [30]. Samples were cleared at 2200 rpm for 20 min and 140 μl of supernatant medium was used for RNA extraction using QIAamp Viral RNA Mini Kits (Qiagen, Venlo, The Netherlands).

### Rotavirus detection

Prior to the RT-PCR, double-stranded RNA was denatured at 95 °C for 2 min followed by cooling on ice. Detection and sequencing PCRs were performed using the Qiagen one-step RT-PCR kit (Qiagen). RVA positivity was detected by real-time RT-PCR targeting the VP6 gene [13] and/or by the conventional PCR targeting the NSP4 gene [25]. RVD was detected by a real-time RT-PCR targeting the VP6 gene [13]. Suboptimal probe binding due to frequent mutations at the binding sites were suspected based on low fluorescence signals in both real-time RT-PCRs and detection was therefore confirmed by gel electrophoresis. The Avian RVA (cell culture supernatant, strain RVA/Chicken-tc/DEU/02V0002G3/2002/G19P[30]) and RVD (intestinal content, strain RVD/Chicken-wt/NLD/10 V0133/2010/GXP[X]) strains, kindly provided by Dr. P. Otto (Friedrich-Loeffler Institute, Germany), served as positive controls.

### Sequencing and genotype characterization

For sequencing of RVA and RVD positive samples, additional primers were designed and evaluated with Geneious software (version 7.1.7; Biomatters Limited; Auckland, New Zealand [http://www.geneious.com]) [31] and Primer3Plus (http://primer3plus.com/cgi-bin/dev/primer3plus.cgi) for partial amplification of VP4, VP6, VP7 of RVA and VP6 and VP7 of RVD (Additional file 1). NSP4 sequences were obtained by using the detection primers [25]. Amplification conditions were as follows: 50 °C for 30 min, 94 °C for 15 min with subsequent 40 cycles of denaturation at 94 °C for 30 s, annealing at 53 °C for 30 s, extension at 72 °C for 60 s. RT-PCR was performed with 3 μl of RNA and 22 μl of PCR mix (containing 1× Qiagen OneStep RT-PCR Buffer, 1.25 mM MgCl_2_, dNTPs at 400 μM each, 1 μl of Qiagen OneStep RT-PCR Enzyme Mix, 0.5 μM of each primer and PCR-grade H_2_O to adjust the final volume). Positive samples were identified by gel-electrophoresis and amplicons of the appropriate size excised from the gel and purified with the QIAquick gel extraction kit (Qiagen). Specific PCR products were directly purified using the JetQuick™ extraction kit (Genomed, Löhne, Germany). Sequencing was performed using the BigDye terminator kit (Applied Biosystems, Foster City, CA) on an ABI 3130 sequencer (Applied Biosystems). RVA genotype classification of partial gene sequences was done as recommended by RCWG [24, 32].

### Phylogenetic analysis

**Fig. 1:**
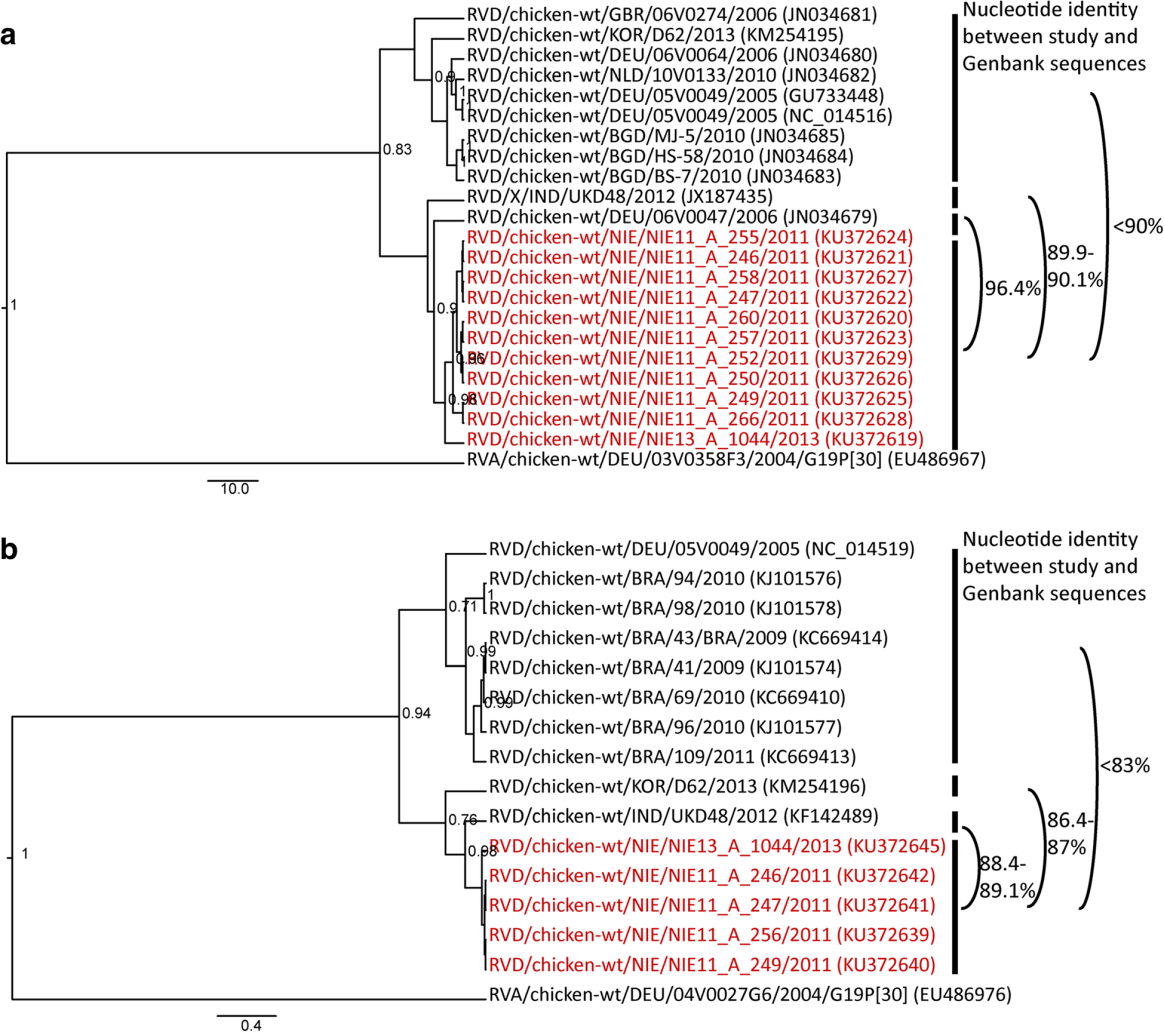
Maximum clade credibility trees of (**a**) VP6 and (**b**) VP7 sequences of RVD. Bayesian analyses of alignments (888 bp for VP6, 637 bp for VP7) comprising unique VP6 and VP7 sequences from GenBank and RVD sequences from this study. Tree topology was tested by posterior probability (pp) and only the well supported values are shown (pp > 0.7). The RVD strains are represented by the official RV nomenclature and the GenBank accession numbers are put in brackets. The study sequences are in red. For each cluster, percentage values of the nucleotide identity shared with the RVD strains from this study are shown

**Fig. 2:**
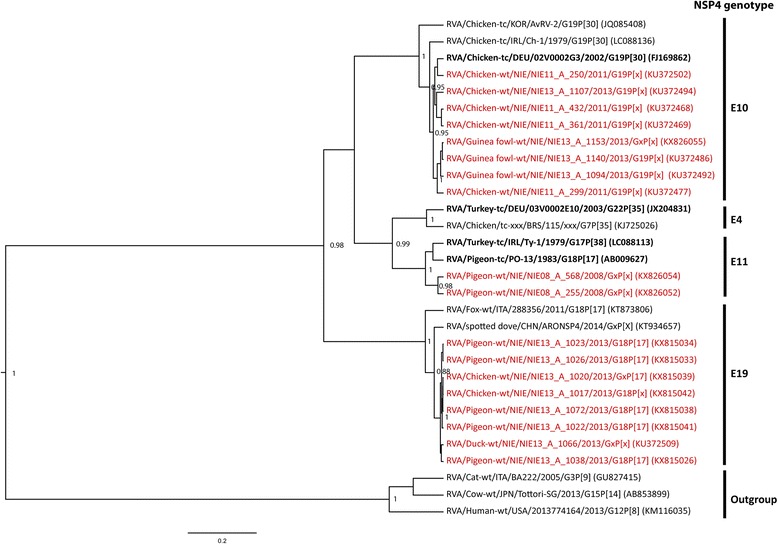
Maximum clade credibility tree of NSP4 sequences of RVA. Bayesian analyses of alignments (529 bp) comprising unique sequences of recognized NSP4 genotypes and sequences identified in this study. Tree topology was tested by posterior probability (pp) and only the well supported values are shown (pp > 0.7). The RVA strains are represented by the official RV nomenclature and the GenBank accession numbers are put in brackets. The study sequences are in red and reference sequences displayed in bold. Genotypes were assigned using the nucleotide cut-off values defined before (19)

**Fig. 3:**
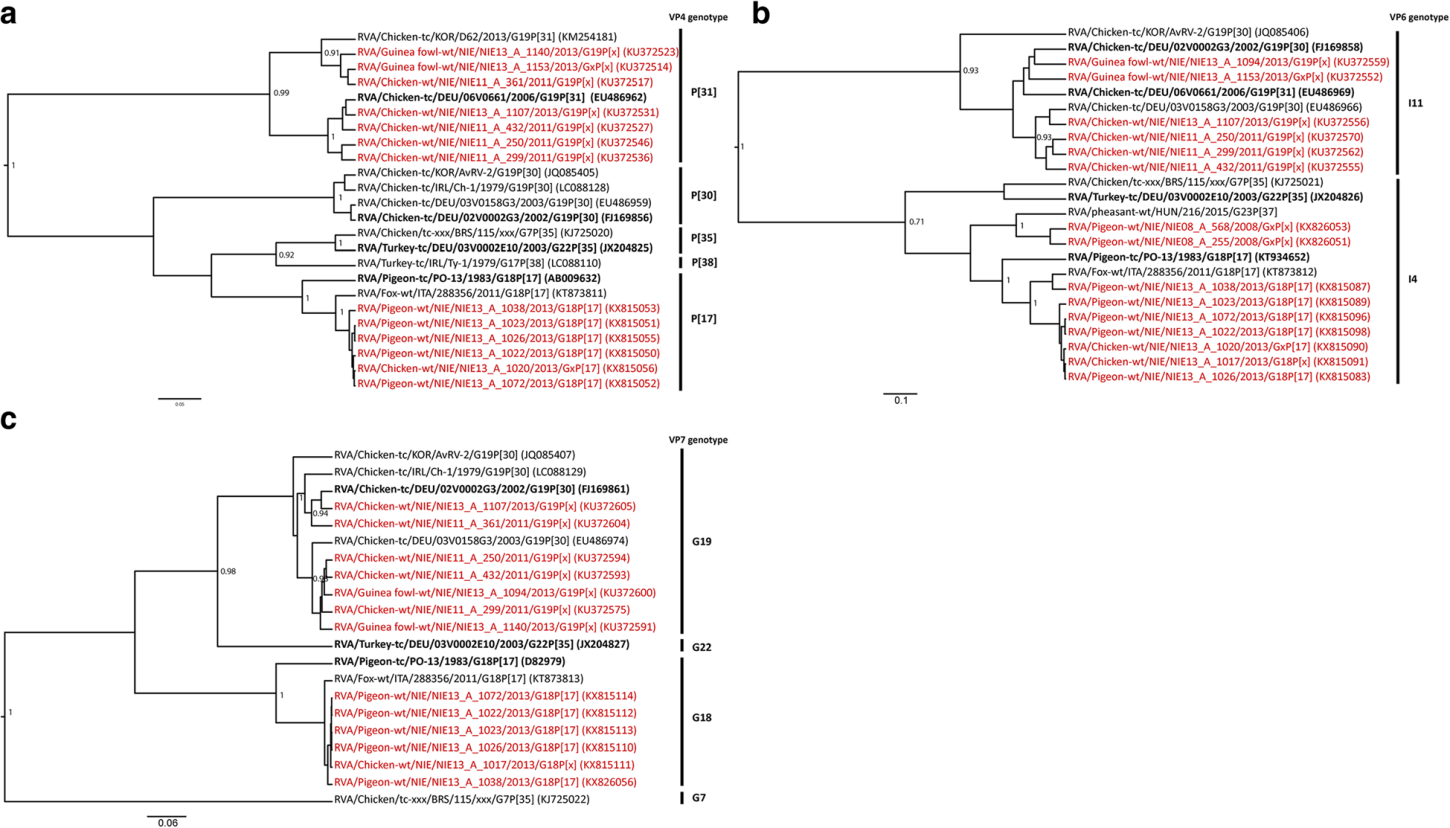
Maximum clade credibility trees of (**a**) VP4, (**b**) VP6 and (**c**) VP7 sequences of RVA. Bayesian analyses of alignments (424 bp for VP4, 1098 bp for VP6, 699 bp for VP7) comprising unique sequences of recognized VP4, VP6 and VP7 genotypes and sequences identified in this study. Tree topology was tested by posterior probability (pp) and only the well supported values are shown (pp > 0.7). The RVA strains are represented by the official RV nomenclature and the GenBank accession numbers are put in brackets. The study sequences are in red and reference sequences displayed in bold. Genotypes were assigned using the nucleotide cut-off values defined before (19)

RV sequences were assembled in Geneious software [31] and trimmed before further analysis. RV sequences from this study were compared to all avian RV sequences available in GenBank database (http://www.ncbi.nlm.nih.gov/Genbank/index.html). Alignments were obtained by applying the ClustalW multiple alignment method (EMBL; Heidelberg, Germany) and poorly aligned regions trimmed using Gblocks as implemented in SeaView software (version 4; CNRS; Villeurbanne, France [http://doua.prabi.fr/software/seaview]) [33–35]. For each alignment, the best-fit model of nucleotide substitution was selected using JModeltest (https://github.com/ddarriba/jmodeltest2) [36] (Additional file 2). Bayesian analyses were applied as statistical inference methods of the phylogenetic analysis, as described before [37]. Representative isolates for which sequences from several genes were obtained or that were detected in new RV host species are shown in Figs. 1, 2 and 3. Additional phylogenetic trees based on all long sequences from this study and including also more GenBank sequences can be found in the supplementary material (Additional files 3, 4, 5 and 6). Only posterior probability values >0.7 are depicted in the phylogenetic trees.

### Statistical analyses

Statistical analyses were performed in R software (version 3.1.0.; R Foundation for Statistical Computing, Vienna, Austria [https://www.r-project.org/]) [38]. Two-sided Fisher’s exact test was applied to identify potential factors influencing virus shedding in chickens: age group, symptoms (diarrhea, increased mortality or no overt symptoms) and RVA-RVD-coinfection (yes/no). Logistic regression analysis was applied to predict whether RVA shedding is affected by age group and RVA-RVD-coinfection. The overall significance of the models was assessed by chi-squared tests and the individual effect of the categorical factors of the model by Wald tests. The packages aod [39] and Rcpp [40] in R were used to test for association between the categorical variables and RV shedding.

## Results

### Rotavirus shedding

In total, 36.1% (126/349, 95% CI: 31–41%) and 31.8% (111/349, 95% CI: 27–37%) of all fecal samples were positive for avian RVA and RVD (Table 1). RVA and RVD co-infection was revealed for 15.2% (53/349, 95% CI: 12–19%) of the birds. RVD shedding among chickens was increased in farms with more than 10,000 birds (*p* = 0.04) and significantly higher shedding rates were observed in Oyo than in Ogun state (41.3 and 30.5%; *p* = 0.007). There was no association between RVA shedding among chickens and collection site or state. Mostly asymptomatic chickens shed RVs. When taking into account only the chickens for which the health status was recorded (*n* = 225), only 18.9% (28/148) of the RVA positive chickens and only 29.7% (44/148) of the RVD positive chickens and were symptomatic. Thus symptomatic chicken were significantly less likely to shed RVA and RVD (Odds Ratio = 0.2 and 0.3, 95% CI = 0.1–0.4 and 0.3–0.8, *p* < 0.001 and 0.01). Logistic regression analysis predicted that RVA positivity is affected by age and RVD coinfection (*p* = 0.002). RVA shedding was significantly increased in chickens that shed also RVD (Odds Ratio = 2.4, 95% CI = 1.5–4.6, *p* = 0.004). Although the overall effect of age group was significant in the Wald test (χ2 = 39.9, degrees of freedom = 3, *p* < 0.001), a significant difference between age groups could only be revealed for age groups 1 and 2 (χ2 = 5.0, degrees of freedom = 1, *p* = 0.025). Chickens aged 26–140 days were more likely to shed RVA than 1–25 days old chickens (Odds Ratio = 1.11, 95% CI = 0.57–2.16).

Besides chickens, other bird species were also positive for RVA (i.e. 23/25 guinea fowls, 13/19 pigeons, 2/16 quails, 5/24 ducks and 1/10 turkeys) and RVD (i.e. 3/25 guinea fowls, 11/19 pigeons, 3/16 quails, 1/24 ducks and 1/10 turkeys). The majority (63.8%, 60/94) of those non-chicken birds were sampled at live bird markets.

### Sequence analysis

Sequencing success was generally low and dependent on host species, RV group and genome segment. This was most probably due to poor sample quality, low copy numbers in fecal samples and/or the low specificity of primers designed on the basis of only a few avian RV sequences available in public databases. Nevertheless, sequencing data was obtained from 69% (87/126) of RVA (Table 2) and 100% (111/111) of RVD positive samples.

**Table 2:** Genetic composition of study sequences

Year	State	Area	Collection site	Bird species	Animal Code	RVA gene			
						VP7	VP4	VP6	NSP4
						Genotype	Genotype	Genotype	Genotype
**2011**	Ogun	Owode-Egba	Farm type 3	Chicken	NIE11_A_255	G19^a^	P[31]^a^	I11	E10
					NIE11_A_252	G19^a^	P[31]^a^	I11	E10
					NIE11_A_254	G19	P[31]^a^	I11	E10
					NIE11_A_242	G19^a^	P[31]^a^	I11	E10
					NIE11_A_251	G19^a^	P[31]^a^	I11	E10
					NIE11_A_253	G19	P[31]^a^	I11	E10
					NIE11_A_256	G19	P[31]^a^	I11	E10^a^
					NIE11_A_257	G19	P[31]^a^	I11	E10
					NIE11_A_260	G19	P[31]^a^	I11	E10
					NIE11_A_250	**G19**	**P[31]** ^**a**^	**I11**	**E10**
					NIE11_A_249	G19	P[31]^a^	I11	E10
					NIE11_A_247	G19^a^	P[31]^a^	I11^a^	E10
					NIE11_A_246	G19^a^	P[31]^a^	I11^a^	E10
					NIE11_A_266	G19^a^	P[31]^a^	I11	n.s.
					NIE11_A_258	G19^a^	P[31]^a^	I11	n.s.
		Ajebo	Farm type 2	Chicken	NIE11_A_461	n.s.	n.s.	n.s.	E10
		Ijebu-Ode	Farm type 3	Chicken	NIE11_A_450	G19	P[31]^a^	I11	E10
					NIE11_A_441	G19	P[31]^a^	I11	n.s.
	Oyo	Ibadan North	Farm type 2	Chicken	NIE11_A_299	**G19**	**P[31]** ^**a**^	**I11**	**E10**
					NIE11_A_295	G19	P[31]^a^	I11	E10
					NIE11_A_300	G19	P[31]^a^	I11	n.s.
					NIE11_A_298	G19	P[31]^a^	I11^a^	n.s.
		Ilora	Farm type 2	Chicken	NIE11_A_365	n.s.	n.s.	n.s.	E10
					NIE11_A_369	n.s.	n.s.	n.s.	E10
					NIE11_A_366	G19	P[31]^a^	I11^a^	E10
					NIE11_A_362	G19	P[31]^a^	I11^a^	E10
					NIE11_A_361	**G19**	**P[31]** ^**a**^	I11^a^	**E10**
					NIE11_A_363	n.s.	n.s.	n.s.	E10
					NIE11_A_436	n.s.	n.s.	n.s.	E10
			Farm type 1	Chicken	NIE11_A_432	**G19**	**P[31]** ^**a**^	**I11**	**E10**
**2013**	Ogun	Adatan	Live bird market	Chicken	NIE13_A_1134	G19	P[31]^a^	n.s.	E10
					NIE13_A_1136	G19	n.s.	n.s.	E10
					NIE13_A_1138	G19	P[31]^a^	n.s.	n.s.
		Ago-Ika	Live bird market	Guinea fowl	NIE13_A_1150	G19	P[31]^a^	n.s.	E10
					NIE13_A_1143	G19	P[31]^a^	n.s.	E10
					NIE13_A_1140	**G19**	**P[31]** ^**a**^	n.s.	**E10**
					NIE13_A_1141	G19	P[31]^a^	n.s.	n.s.
					NIE13_A_1145	G19	P[31]^a^	I11	E10
					NIE13_A_1152	G19	P[31]^a^	I11	n.s.
					NIE13_A_1142	G19	P[31]^a^	n.s.	E10
					NIE13_A_1144	G19	P[31]^a^	I11	E10
					NIE13_A_1146	G19	n.s.	n.s.	E10
					NIE13_A_1092	G19	P[31]^a^	I11	E10
					NIE13_A_1095	G19	P[31]^a^	n.s.	E10
					NIE13_A_1094	**G19**	**P[31]** ^**a**^	**I11**	**E10**
					NIE13_A_1096	G19	P[31]^a^	I11	E10
					NIE13_A_1149	G19	P[31]^a^	I11	n.s.
					NIE13_A_1093	G19	P[31]^a^	I11	E10
					NIE13_A_1147	G19	n.s.	I11	E10
					NIE13_A_1091	G19	P[31]^a^	I11	E10
					NIE13_A_1153	G19^a^	**P[31]** ^**a**^	**I11**	**E10**
				Duck	NIE13_A_1066 ^b^	n.s.	**P[31]** ^**a**^	n.s.	**E19**
		Lafenwa	Backyard farm	Chicken	NIE13_A_1104	n.s.	P[31]^a^	n.s.	E10
					NIE13_A_1107	**G19**	**P[31]** ^**a**^	**I11**	**E10**
		Alabata	Farm type 2	Chicken	NIE13_A_1040	G19	P[31]^a^	n.s.	E10
		Itoku	Live bird market	Guinea fowl	NIE13_A_1008 ^b^	G19	P[31]^a^	n.s.	E19
		Ewekoro	Farm type 1	Chicken	NIE13_A_1013	n.s.	n.s.	n.s.	E19
					NIE13_A_1017	**G18**	P[17]^a^	**I4**	**E19**
					NIE13_A_1019	n.s.	n.s.	I4	E19^a^
					NIE13_A_1020	n.s.	**P[17]** ^**a**^	**I4**	**E19**
		Abeokuta north	Live bird market	Pigeon	NIE13_A_1022	**G18**	**P[17]**	**I4**	**E19**
					NIE13_A_1023	**G18**	**P[17]**	**I4**	**E19**
					NIE13_A_1024	n.s.	n.s.	I4	E19
					NIE13_A_1025	G18	P[17]	I4	E19
					NIE13_A_1026	**G18**	**P[17]**	**I4**	**E19**
					NIE13_A_1128	n.s.	n.s.	I4	E19
					NIE13_A_1129	n.s.	P[17]^a^	I4	E19
				Duck	NIE13_A_1068	n.s.	n.s.	I4	E19^a^
		Odeda	Farm type 1	Chicken	NIE13_A_1028	n.s.	n.s.	I4^a^	E19
					NIE13_A_1169 ^**b**^	n.s.	P[17]^a^	I11	E10
			Farm type 2	Chicken	NIE13_A_1039	n.s.	n.s.	I4	E19
					NIE13_A_1045 ^b^	G19^a^	n.s.	I4^a^	E10
					NIE13_A_1047	n.s.	n.s.	I4^a^	E19
		Abeokuta south	Live bird market	Pigeon	NIE13_A_1036	G18	P[17]^a^	I4	E19
					NIE13_A_1037 ^b^	G19^a^	n.s.	I4	E19
					NIE13_A_1038	**G18**	**P[17]**	**I4**	**E19**
				Duck	NIE13_A_1164	n.s.	n.s.	n.s.	E19
				Chicken	NIE13_A_1081	n.s.	n.s.	n.s.	E19
					NIE13_A_1085	n.s.	n.s.	I4	E19
					NIE13_A_1138 ^b^	n.s.	P[17]^a^	n.s.	E10
			Backyard farm	Pigeon	NIE13_A_1072	**G18**	**P[17]**	**I4**	**E19**
					NIE13_A_1074	n.s.	n.s.	I4^a^	E19
					NIE13_A_1075 ^b^	G19^a^	n.s.	I4^a^	E19
	Oyo	Akinyele	Live bird market	Guinea Fowl	NIE13_A_1069	n.s.	n.s.	I4^a^	E19
					NIE13_A_1070	n.s.	n.s.	I4^a^	E19
					NIE13_A_1071	n.s.	n.s.	I4^a^	E19
		Ibadan North	Live bird market	Quail	NIE13_A_1119	n.s.	n.s.	I4^a^	E19

*n.s*. no sequence

^a^ Sequence too short for definite genotype allocation using the criteria of the RCWG

^b^ Potential reassortants

**Bold face** sequences are shown in phylogenetic tree of Figs. 2 and 3

RVD sequences showed little diversity (>98% nucleotide identity for VP6 and >99% nucleotide identity for VP7) and were clearly distinct from RVA (Fig. 1). Both phylogenetic and nucleotide identity analyses of partial RVD sequences showed that all VP6 sequences grouped into two major clusters (Fig. 1a) with less than 90% nucleotide identity. Similarly, partial VP7 sequences grouped into two major clusters with less than 83% nucleotide identity (Fig. 1b).

Genotype assignment of RVA can be retrieved from Table 2. In general, RVA strains from the same place and time point had highly similar sequences. On the phylogenetic tree of NSP4, the first diverging event led to two well supported lineages: one comprising the avian and the other the mammalian RVA strains (Fig. 2). All avian NSP4 strains fell into four clusters, corresponding to four distinct NSP4 genotypes (Fig. 2, Additional file 3). Genotype E10 (reference strain RVA/Chicken-tc/DEU/02V0002G3/2002/G19P[30]) regrouped most of our sequences from chickens and guinea fowls. The remaining sequences from various bird species (including pigeons, ducks, chickens, guinea fowls and quail) were closely related to two fox and dove sequences (strains: RVA/Fox-tc/ITA/288356/2011/G18P[17] and RVA/spotted dove/CHN/ARONSP4/2014/GxP[X]). Those sequences were assigned to the new NSP4 genotype, E19 (Table 2).

Most partial VP4 sequences from chickens and guinea fowls clustered together with RVA/Chicken-tc/DEU/06 V0661/2006/G19P[31] within genotype P[31] (Fig. 3a; Additional file 3). Similar to NSP4, all pigeon and duck sequences and a few chicken and guinea fowl sequences clustered together and were assigned to genotype P[17], that includes also the above fox strain and the reference strain RVA/Pigeon-tc/PO-13/1983/G18P[17]. The phylogenetic trees of NSP4 and VP4 had topologies similar to those obtained for VP6 and VP7 (Figs. 2 and 3; Additional files 5 and 6). Most chicken and guinea fowl strains were assigned to VP6 genotype I11 and VP7 genotype G19. The remaining strains were closely related to the fox and the pigeon PO-13 strains and assigned to VP6 genotype I4 and VP7 genotype G18 (Fig. 3b and c).

Thus, the majority of our sequences had one of the following two genotype constellations: G19-P[31]-I11-E10 or G18-P[17]-I4-E19. However, 7/87 samples showed different genome constellations possibly indicative of genetic reassortment (Table 2).

## Discussion

In Nigeria, several viral diseases affect productivity and economic success of the poultry industry [41–43]. In this study we show that RV infections among different bird species may constitute an additional threat. High levels of RVA and RVD shedding were detected in species such as guinea fowls and ducks, for which genetic and epidemiological data on RVs are largely missing. However, the effect of RV infection on animal health seemed limited as mostly asymptomatic birds were found to shed RVs. This is in line with previous studies that reported subclinical RV infections mostly in adult birds [4, 7]. Subclinical shedding contributes considerably to the maintenance of a high viral contamination in the farm and environment that may, in turn, be an important source of infection for younger animals. Growth performance and egg production may also suffer, in particular in large farms (>500 animals) [27].

The genetic diversity of the Nigerian RV strains was high. The Nigerian sequences considerably extend the current RV sequence database and will be useful to increase specificity of the molecular detection assays. We showed that RVD is not solely shed by chickens, but also by pigeons and guinea fowls from which partial sequences were obtained (e.g. GenBank accession numbers KU372630 and KX907137). Phylogenetic tree topology of partial gene sequences suggests at least two separate lineages of VP6 and VP7 of RVD (Fig. 1). Full length RVD sequences of these and other genes will be required to define genotyping criteria.

Similarly, RVA was found in bird species other than chickens. Most guinea fowls sampled at live bird markets were infected by the same strains as chickens (G19-P[31]-I11-E10). Also G18-P[17]-I4-E19 was found in pigeons, chickens (Figs. 2 and 3), guinea fowls, quails and ducks (Additional file 3), suggesting host permissiveness of these strains when different bird species are mixed, for instance at live bird markets. In this context, it is interesting that avian RVA G18-P[17]-I4-E19 strains were distantly related to the other typical chicken RVs, and closely related to a fox RV strain only recently published in GenBank. Phylogenetic analyses revealed early divergence between mammalian and avian RVA strains [18] and only few inconclusive examples of mammalian-bird transmission of RVA have been reported [44–46]. It remains elusive whether the RVA strain originated from an infected fox or from an infected bird prey.

The G18-P[17]-I4-E19 strains were frequently found in pigeons in our study but also in a Spotted dove (*Streptopelia chinensis*) in China (NSP4 only) and in a pigeon (VP4, VP6, VP7) back in 1983 [46]. To investigate whether Columbidae are the principal reservoir of this group of RVA strains, we screened, with the same approach, fecal samples of wild pigeons (*n* = 161) collected in Nigeria in 2008 (data not shown). The NSP4 fragments obtained from a *Streptopelia roseogrisea* and a *Turtur abyssinicus* were assigned to another genotype, E11 and, the VP6 fragments, although assigned to genotype I4, were most similar to a recently published pheasant strain (Figs. 2 and 3b), and not to the fox strain. Thus, no statement can be made on the principal reservoir of G18-P[17]-I4-E19.

Consistent with previous studies [13, 16, 18, 47–49], we found a clearly separated and independent evolution of RVA and RVD strains (Figs. 1, 2 and 3) and a high proportion of RVA/RVD co-infections. RVA/RVD co-infections may lead to natural cross-group reassortment, which may be further facilitated by identical gene termini [18, 20]. Natural RVA-RVD cross-group reassortants have not yet been described and we found only preliminary indications of reassortment between RVA strains among our sequences. Definite characterization of such reassortants would necessitate virus isolation and full genome sequencing. Current reverse genetic approaches for RVA [50] will facilitate the rescue of reassortants in vitro and evaluation of the consequence of natural co-infections on virus evolution and clinical outcome.

## Conclusion

We show that co-circulation of diverse RV strains in mixed bird farms and at live bird markets promotes interspecies transmission. The study stresses once more that rearing and trading conditions in rural Africa favor emergence of novel viruses with low host-specificity and with only partly understood economic and clinical impact.

## Additional files

Additional file 1: Primers used for the detection of avian group A and D rotaviruses by real-time RT-PCR and for amplification of segments of VP4, VP6, VP7 and NSP4 for sequencing. (PDF 198 kb)

Additional file 2: Best-fit models of nucleotide substitution that were selected for each phylogenetic analysis using JModeltest (https://github.com/ddarriba/jmodeltest2). (PDF 167 kb)

Additional file 3: Maximum clade credibility tree of partial NSP4 gene sequences of Rotavirus group A (RVA). (PDF 279 kb)

Additional file 4: Maximum clade credibility tree of partial VP4 gene sequences of Rotavirus group A (RVA). (PDF 288 kb)

Additional file 5: Maximum clade credibility tree of partial VP6 gene sequences of Rotavirus group A (RVA). (PDF 312 kb)

Additional file 6: Maximum clade credibility tree of partial VP7 gene sequences of Rotavirus group A (RVA). (PDF 305 kb)

## Abbreviations

ICTV: International Committee on Taxonomy of Viruses
NSP: Nonstructural proteins
RCWG: Rotavirus Classification Working Group
RV: Rotavirus
RVA-RVH: Rotavirus groups A to H
RVs: Rotaviruses
