# Supplementary material for: Molecular epidemiology of Avian Rotaviruses Group A and D shed by different bird species in Nigeria
Source: Virol J. 2017 Jun 12;14:111. doi: 10.1186/s12985-017-0778-5 (PMC5469043; doi:10.1186/s12985-017-0778-5)
Supplement: Supplementary file 1 — Primers used for the detection of avian group A and D rotaviruses by real-time RT-PCR and for amplification of segments of VP4, VP6, VP7 and NSP4 for sequencing. (PDF 198 kb) [file 12985_2017_778_MOESM1_ESM.pdf]

Additional File 1: Primers used for the detection of avian group A and D rotaviruses by real-time RT-PCR and for amplification of segments of VP4, VP6, VP7 and NSP4 for sequencing.

Rotavirus group	Target segment	Target gene	Primer sense	Primer sequence (5`-3`)	Amplicon size (bp)	Reference
**Detection**						
Rotavirus A	Seg 6	VP6	F	GAGCAACTATTGATTACTTCATTGA	114	(1)
			R	AAAGTTGCCTTARTGCATTAGA		
			Probed	AGGAGCTATTCCATTACGTTGAGATTC		
Rotavirus D	Seg 6	VP6	F	GCGACAACTGAGACAACTG	186	(1)
			R	GGAAGCAGTTGTCATCAAC		
			Probed	TTGCATATTAGATTGTCTCGCTGGTGTATA		
**Sequencing**						
Rotavirus A	Seg 10	NSP4	F	GTGCGGAAAGATGGAGAAC	630	(2)
			R	GTTGGGGTACCAGGGATTAA		
	Seg 6	VP6	F	CGAAGTCTTCATCATGGAT	420	this study
			R	RCCTGTRCGTTGYCTTCTGTT		
	Seg 6	VP6	F	GTAATGGAATWGCDCCNCAATC	777	(3)
			R	ATACCTGSWGGAAAWACTGGTCC		
	Seg 6	VP6	F	GCAGCACCATTTCCWAATCAT	353	this study
			R	GGTCACATCCTCTCACTATA		
	Seg 9	VP7	F	GAATGGCTAGCTCATATGTT	487	this study
			R	TGTCTGAGTATTTARTGGGCATA		
	Seg 9	VP7	F	CAGACTTCAGAGGCTAATAA	378	this study
			R	GCGCGATCTTCTAGACATA		
	Seg 9	VP7	F	GCTAATTCGCCCACTCTTTGC	this study	
			R	CCCTGCCCATTGCTATCCAT		

	Seg 9	VP7	F	GAATGGAAAAGCACGGTGGC		this study
			R	GCACTCGCGTTAAGTGATCG		
	Seg 4	VP4	F	GGCTATAAAATGGCTTCDCTC	645	this study
			R	TGWATTGGAGGYAGACCATT		
	Seg 4	VP4	F	GAACAGTGCCAAATGTRCAA	733	this study
			R	CGAATGATGGCTCACCAA		
	Seg 4	VP4	F	GCTGGTAAATTATATGCATATAG	524	this study
			R	TGCTTGTGAATCATCCCAGTA		
	Seg 4	VP4	F	AGACAACTTCTAGCAAAYTCRTACACT	512	this study
			R	CCATGATATACRTACAACCKTCCTTG		
	Seg 4	VP4	F	ATGCTTTYATGAAAGAYCAAGGA	690	this study
			R	CATTTGTGGCCATKGYCCAA		
	Seg 4	VP4	F	ATGTACGATCGCTAARTGCAGAA	780	this study
			R	TCCACCTTCAGTCTGYGTWGC		
	Seg 4	VP4	F	GACATTGCCACDCAAACWTCA	450	this study
			R	AATCTTTCAGCACWCKTGGRTC		
Rotavirus D	Seg 6	VP6	F	CCAACGTRTCAGATGTRAT	491	this study
			R	GCTGYCCACGAACAAAGTCYT		
	Seg 6	VP6	F	CTACTGGTGGATTGGCTACTA	362	this study
			R	GCAAATCKKGTTCCTGAYACATT		
	Seg 6	VP6	F	GAATTYCAACTTGCAGGACAA	485	this study
			R	CTGCTGACGTATGATAACAGATT		
	Seg 6	VP6	F	GCTGGAAATCCATCAGCWA	322	this study
			R	GCAGATATTACTCCGCATT		
	Seg 6	VP6	F	GGCTCTAAGTACATTTCAGAATGG	740	this study
			R	CCAGGAGGGAAAACTGGTCC		
	Seg 9	VP7	F	GGAGTGACGCACCTGTATGA	552	this study
			R	GCGTTTGTTGATTCAATGGGC		
	Seg 9	VP7	F	CATCAATGGATGAAATAATAACAGG	372	this study
			R	AGCGACATTRGTTCTCTCGCTC		

4

References

1. **Otto PH, Ahmed MU, Hotzel H, Machnowska P, Reetz J, Roth B, Trojnar E, Johne R.** 2012. Detection of avian rotaviruses of groups A, D, F and G in diseased chickens and turkeys from Europe and Bangladesh. Vet Microbiol **156:**8-15.
2. **Pantin-Jackwood MJ, Day JM, Jackwood MW, Spackman E.** 2008. Enteric viruses detected by molecular methods in commercial chicken and turkey flocks in the United States between 2005 and 2006. Avian Dis **52:**235-244.
3. **Schumann T, Hotzel H, Otto P, Johne R.** 2009. Evidence of interspecies transmission and reassortment among avian group A rotaviruses. Virology **386:**334-343.
