# Supplementary material for: Molecular epidemiology of Avian Rotaviruses Group A and D shed by different bird species in Nigeria
Source: Virol J. 2017 Jun 12;14:111. doi: 10.1186/s12985-017-0778-5 (PMC5469043; doi:10.1186/s12985-017-0778-5)
Supplement: Supplementary file 2 — Best-fit models of nucleotide substitution that were selected for each phylogenetic analysis using JModeltest (https://github.com/ddarriba/jmodeltest2). (PDF 167 kb) [file 12985_2017_778_MOESM2_ESM.pdf]

Additional File 2: Best-fit models of nucleotide substitution that were selected for each phylogenetic analysis using JModeltest (http://jmodeltest.org/user/dashboard).

Phylogenetic analyses of:	Alignment length	nucleotide substitution model selected	Figure in manuscript
VP6 gene of Rotavirus D	888	HKY + G	Figure 1
VP7 gene of Rotavirus D	637	HKY + G	Figure 1
NSP4 gene of Rotavirus A	529	HKY + G	Figure 2
VP4 gene of Rotavirus A	424	GTR + G	Figure 3
VP6 gene of Rotavirus A	1098	HKY + I + G	Figure 3
VP7 gene of Rotavirus A	699	GTR + I + G	Figure 3
NSP4 gene of Rotavirus A, all study sequences	529	HKY + G	Supplementary Figure 1
VP4 gene of Rotavirus A, all study sequences	424	GTR + G	Supplementary Figure 2
VP6 gene of Rotavirus A, all study sequences	1098	HKY + I + G	Supplementary Figure 3
VP7 gene of Rotavirus A, all study sequences	699	GTR + I + G	Supplementary Figure 4
