# Supplementary material for: Molecular epidemiology of Avian Rotaviruses Group A and D shed by different bird species in Nigeria
Source: Virol J. 2017 Jun 12;14:111. doi: 10.1186/s12985-017-0778-5 (PMC5469043; doi:10.1186/s12985-017-0778-5)
Supplement: Supplementary file 4 — Maximum clade credibility tree of partial VP4 gene sequences of Rotavirus group A (RVA). (PDF 288 kb) [file 12985_2017_778_MOESM4_ESM.pdf]

Additional File 4

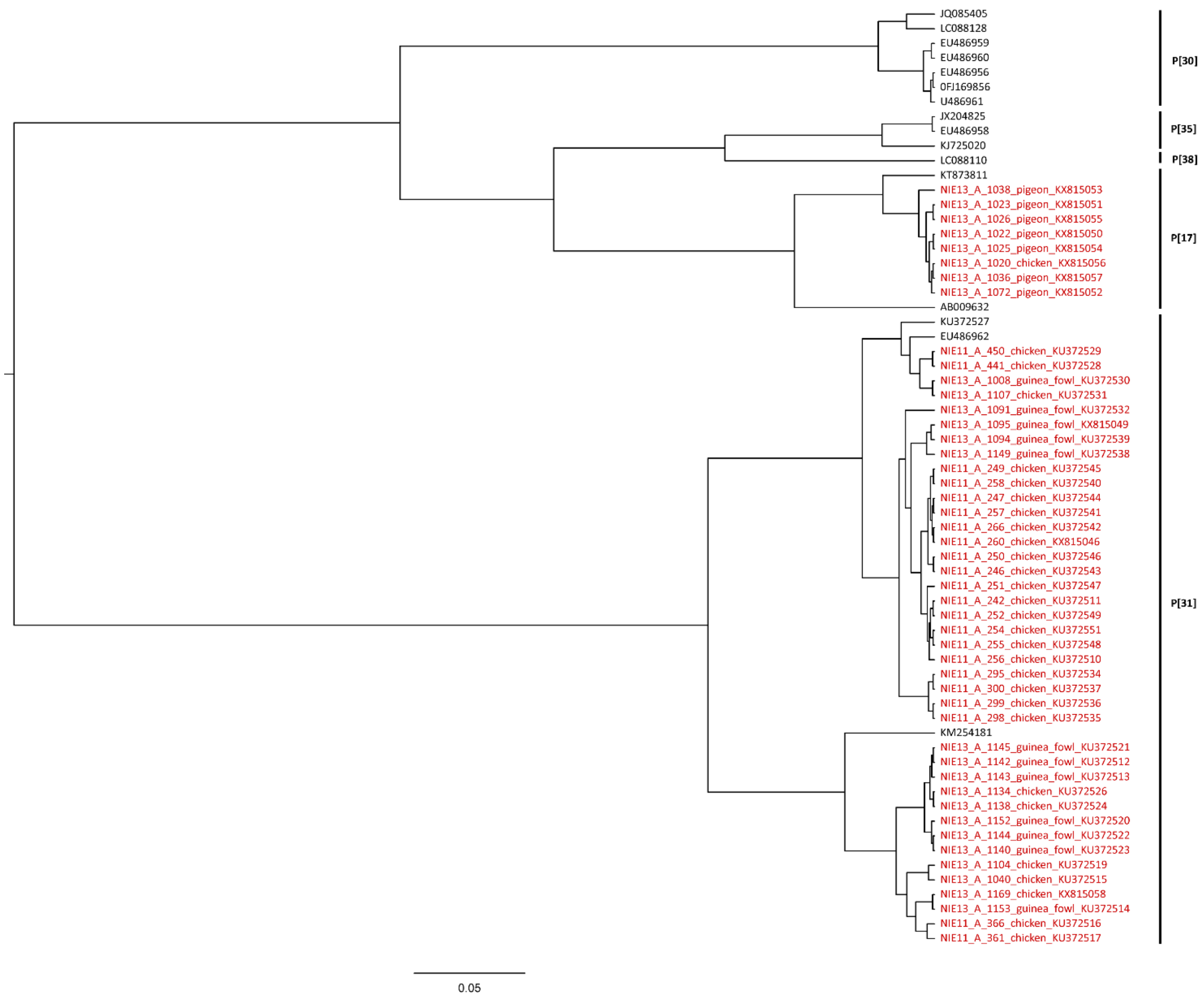

Maximum clade credibility tree of partial VP4 gene sequences of Rotavirus group A (RVA). Bayesian analyses of alignments (424 bp) comprising sequences of all available VP4 sequences that are available in Genbank (http://www.ncbi.nlm.nih.gov/Genbank/index.html), as well as all long sequences identified in this study. Tree topology was tested by posterior probability (pp) and only the well supported values are shown (pp>0.7). The RVA strains are represented by their GenBank accession numbers and study sequences are in red. Genotypes were assigned using the nucleotide cut-off values defined before (1).

References

1. **Matthijnssens J, Ciarlet M, McDonald SM, Attoui H, Banyai K, Brister JR, Buesa J, Esona MD, Estes MK, Gentsch JR, Iturriza-Gomara M, Johne R, Kirkwood CD, Martella V, Mertens PP, Nakagomi O, Parreno V, Rahman M, Ruggeri FM, Saif LJ, Santos N, Steyer A, Taniguchi K, Patton JT, Desselberger U, Van Ranst M.** 2011. Uniformity of rotavirus strain nomenclature proposed by the Rotavirus Classification Working Group (RCWG). Arch Virol **156:**1397-1413.
